# Characterization of tumor microenvironment and sensitive chemotherapy drugs based on cuproptosis-related signatures in renal cell carcinoma

**DOI:** 10.18632/aging.205043

**Published:** 2023-09-19

**Authors:** Jiefu Tang, Fan Yao, Zhiyong Yao, Xiao-Liang Xing

**Affiliations:** 1 The First Affiliated Hospital of Hunan University of Medicine, School of Public Health and Laboratory Medicine, Hunan University of Medicine, Huaihua 418000, Hunan, P.R. China

**Keywords:** RCC, cuproptosis, immunotherapy, chemotherapy, prognosis

## Abstract

Cuproptosis is a novel type of copper-induced cell death and is considered as a new therapeutic target for many cancers. Distant metastases occur in about 40% of patients with advanced renal cell carcinoma (RCC), with a poor 5-year prognosis of about 10%. Through a series of comprehensive analyses, four differentially expressed cuproptosis-related lncRNAs (DECRLs) were identified as candidate biomarkers for RCC. The risk model constructed by using these four DECRLs can better predict the prognosis of patients with RCC, which is determined by the receiver operating characteristic (Time dependent area under curve value at 1-year, 3-year, 5-year, and 10-year were 0.82, 0.80, 0.76, and 0.73 respectively). There were significant differences in immune status between high-risk and low-risk RCC patients. The differentially expressed gene enrichment terms between high- and low-risk patients was also dominated by immune-related terms. The risk score was also correlated with immunotherapy as measured by the tumor immune dysfunction and exclusion (TIDE) score. In addition, we also found that the sensitivity of many chemotherapy drugs varies widely between high- and low-risk patients. The sensitivity of the three chemotherapy drugs (AZD4547, Vincristine, and WEHI-539) varied among high- and low-risk patients, and was significantly negatively correlated with risk values, suggesting that they could be used as clinical treatment drugs for RCC. Our study not only obtained four potential biomarkers, but also provided guidance for immunotherapy and chemotherapy treatment of RCC, as well as new research strategies for the screening of other cancer biomarkers and sensitive drugs.

## INTRODUCTION

Copper is an indispensable element in the human body which participates in many biological processes, including mitochondrial respiration, iron absorption, oxidation resistance, and detoxification [1]. Dysregulation of copper homeostasis may cause many diseases, such as Menkes disease, Wilson disease, and neurodegenerative diseases [2]. High concentrations of copper ions bind to tricarboxylic acid circulating lipoacylated proteins, resulting in abnormal aggregation of lipoacylated proteins and loss of iron-sulfur tuftin, ultimately leading to protein-toxic stress response-mediated cell death, which named cuproptosis [3]. Additionally, recent studies have found that cuproptosis is closely related to tumor cell development, angiogenesis and metastasis [4–7].

Kidney cancer is one of the most common malignancies of the urinary system, causing nearly 430000 new cases and 180000 deaths [8]. Renal cell carcinoma (RCC) is one of the most important kidney cancers with heterogeneity in histology, molecular features, clinical outcome and therapeutic response [9, 10]. Of which, clear cell renal cell carcinoma (KIRC) accounts for about 70–80% of RCC while papillary renal cell carcinoma (KIRP) accounts for about 15–20% of renal cell carcinoma [11–13]. KIRC is characterized by chromosome 3p deletion and VHL gene mutation while KIRP is characterized by trisomy of chromosomes and loss of chromosome 9p [14]. The 5-year overall survival (OS) rates for KIRC and KIRP patients are 55–60% and 80–90%, respectively [15]. However, both KIRC and KIRP originate from cells in the proximal convoluted tubules of the nephron [16]. Early excision is considered the best treatment for kidney cancer [17]. However, up to 40% of patients develop metastases after initial surgical treatment of local renal cell carcinoma, resulting in a poor prognosis (the 5-year survival rate is about 10%) [18, 19]. Cancer metastasis is highly dependent on tricarboxylic acid cycle reprogramming. Copper ion is closely related to tricarboxylic acid cycle, and down-regulating tricarboxylic acid cycle is conducive to tumor invasion [20]. Recent studies have found that targeted therapy combined with immunotherapy has a good therapeutic effect in patients with advanced KIRC, showing a trend of gradually replacing targeted therapy alone [21]. It is interesting to note that previous studies have shown that copper loss caused by tetrathiomolybdate affects the immune response. Copper ion may regulate the expression of PD-L1 and affect the immune escape of tumor [22, 23]. Therefore, it is of great significance to establish new molecular phenotypes to more finely classify advanced or unresectable RCC patients, and to select effective therapeutic drugs for their personalized selection.

As part of our study, we constructed two cuproptosis-related patterns; each associated with different prognostic and tumor microenvironment characteristics. We proposed the use of cuproptosis-related signatures scores to quantify the prognosis and therapeutic response in each RCC patient, based on the expression profile of cuoproptosis related genes and lncRNAs. This scoring model can help clinicians develop more effective and personalized treatment strategies.

## MATERIALS AND METHODS

### Data collection and differentially expressed analysis

RNA sequencing data and clinical information data for RCC was obtained from The Cancer Genome Atlas Program (TCGA) (https://portal.gdc.cancer.gov/) database, which included a database of 601 tumor samples with alive vital and 217 tumor sample with dead vital. DESeq2 in R (3.6.1) was used to normalize the genes expression level and screened the differentially expressed genes with the following criteria basemean ≥ 50, Logfoldchange ≥ 0.5, and *p*adj < 0.05. Cuproptosis-related lncRNAs were determined by Pearson correlation analysis with the following criteria: r ≥ 0.3 and *p* < 0.05.

### Development and validation of prognostic risk assessment

According to the median value of each gene expression, all RCC patients were divided into a high expression group and low expression group. A univariate Cox regression analysis was used to screen survival-related signatures for RCC in the entire group. Kaplan-Meier (K-M) curve was used to diagram the overall survival status.

To find the independent overall survival-related biomarkers and construct the prognostic model of RCC, RCC patients were randomly divided into training and validation groups. The specific clinical information of those patients in each group was shown in Table 1. Multivariate Cox regression analysis was used to screen the independently survival-related signatures for RCC in the training group.

**Table 1:** The feature of RCC patients in different group.

**Feature**		**Training (*n* = 409)**	**Validation (*n* = 409)**	**Entire (*n* = 818)**
OS time (month)		41.08	41.19	41.13
Vital	Alive	293	308	601
	Dead	116	101	217
Age	<50	70	81	151
	≥50	339	328	667
Pathologic tumor (T)	T1	224	240	464
	T2	57	44	101
	T3	121	117	238
	T4	6	7	13
	TX	1	1	2
Pathologic node (*N*)	N0	157	131	288
	N1	21	19	40
	N2	1	3	4
	NX	230	256	486
Pathologic metastasis (M)	M0	257	258	515
	M1	48	39	87
	MX	104	112	216
Pathologic stage (S)	SI	208	229	437
	SII	47	31	78
	SIII	86	88	174
	SIV	52	45	97
	SX	16	16	32

Overall survival related biomarkers obtained by multivariate Cox regression analysis were used to construct and verify the risk model in the training, validation, and entire groups. $\text{Risk score}=\sum_{\text{i}=1}^{\text{n}}\text{Coef}(\text{i})\times \text{Exp}(\text{i}).$ Coef (i) and Expr (i) denote the regression coefficient of the multivariate Cox regression analysis for each lncRNA and normalized expression level for each lncRNA, respectively. Yonden index from the training group was set as the optimal cutoff value.

### PCA, GO, and KEGG analysis

The cuproptosis-related lncRNAs for RCC sample were classified by principal component analysis (PCA) to visualize the distribution with different status.

Gene set variation analysis (GSVA) and Gene Set Enrichment Analysis (GSEA) were used to carry out the Gene Oncology (GO) analysis, including biological process (BP), cellular component (CC), and molecular function (MF), and Kyoto Encyclopedia of Genes and Genomes (KEGG) analysis. *p* < 0.05 was considered as significantly enriched BP, CC, MF, and pathways.

### Tumor immune analysis

ESTIMATE (Estimation of STromal and Immune cells in MAlignant Tumours using Expression data) in R (3.6.1) was used to evaluate the ESTIMATEScore, ImmuneScore, StromalScore, and tumor purity. Single-sample GSEA algorithm was used to evaluate the immune score of different immune cells and factors.

TIMER2.0 (Tumor IMmune Estimation Resource) was used to calculate the immune infiltration profile of immune cells and factors in each RCC patients (http://timer.cistrome.org/).

### Tumor mutation burden and tumor immune dysfunction and exclusion analysis

Somatic mutation data of KIRC and KIRP were downloaded from TCGA database, maftools in R (3.6.1) was used to analysis the tumor mutation burden (TMB). The tumor immune dysfunction and exclusion (TIDE) was estimated through GALAXY of BioInfoTools (http://biowinford.site:3838/OnlineTools4/).

### Chemotherapy drug sensitivity analysis and statistical analysis

Oncopredict algorithm was used to evaluate the sensitivity of different chemotherapy drug in patients with RCC with different status using their differentially expressed genes (http://biowinford.site:3838/OnlineTools4/). A repeated measure ANOVA followed by an unpaired two-tailed student’s *t*-test was used as indicated.

### Availability of data and materials

The data that support the findings of this study are openly available in TCGA at https://portal.gdc.cancer.gov/.

## RESULTS

### Evaluation of cuproptosis-related genes as prognostic biomarkers for RCC

**Figure 1:**
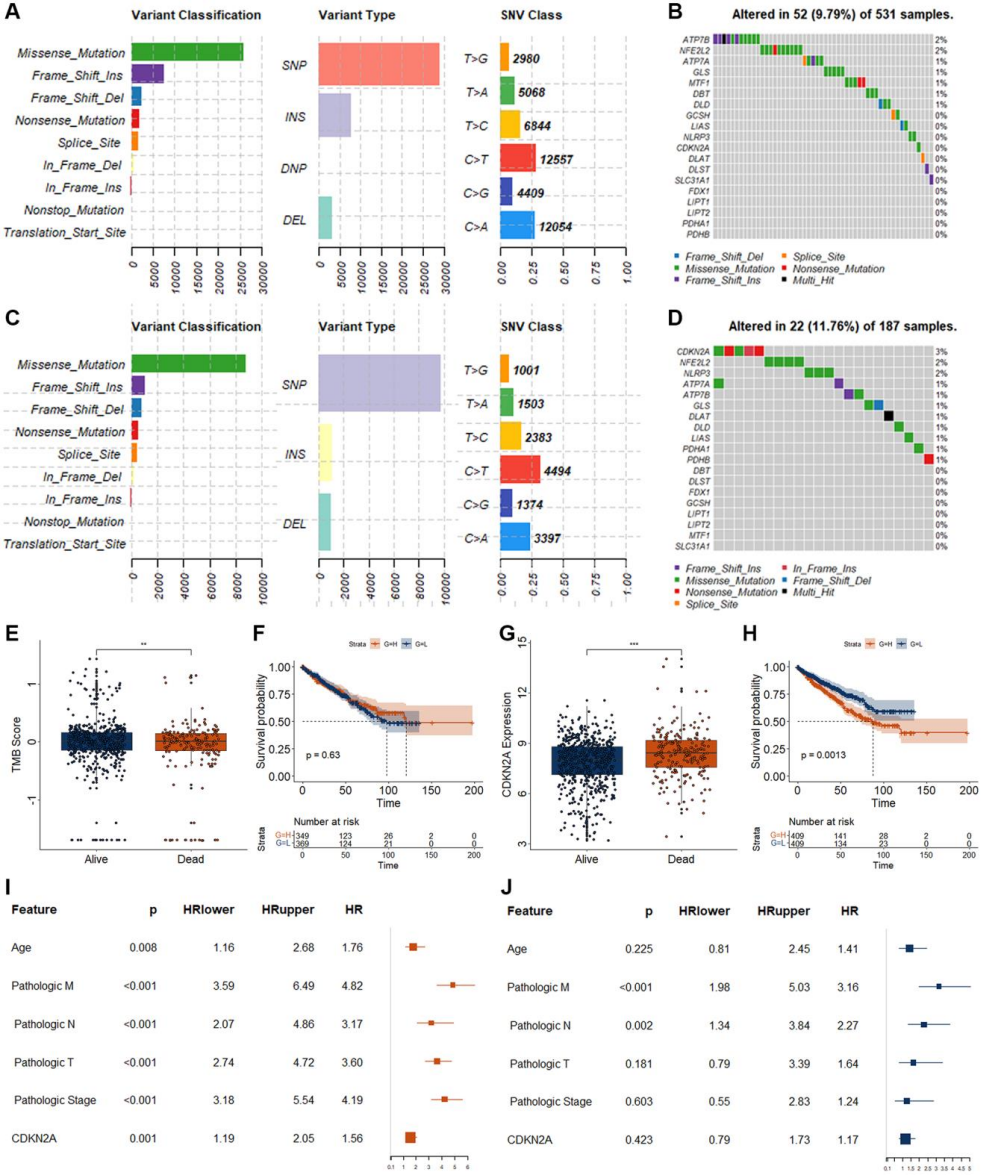
**Genetic variations and biomarker screening of CRGs in RCC.** (**A**) Summary of variation in alive RCC patients. The x axis represents the number of mutations, and the y axis represents the category of mutation. (**B**) Genetic mutations landscape of 19 CRGs in alive RCC patients. (**C**) Summary of variation in dead RCC patients. The x axis represents the number of mutations, and the y axis represents the category of mutation. (**D**) Genetic mutations landscape of 19 CRGs in dead RCC patients. (**E**) Analysis of TMB difference in patients with different survival status of RCC. (**F**) K-M curve for RCC patients with different TMB score. H, represents high TMB group as measured by the median value. L, represents low TMB group as measured by the median value. (**G**) Expression level of CDKN2A in patients with different survival status of RCC. (**F**) K-M curve for RCC patients with different CDKN2A expression level. (**H**) represents high expression group as measured by the median value. L, represents low expression group as measured by the median value. (**I**, **J**) Results of univariate (**I**) and multivariate (**J**) Cox regression for CDKN2A and different clinical features. ^*^p < 0.05. ^**^p < 0.01.

A total of 818 RCC patients (601 alive and 217 dead) with RNAseq data and 718 RCC patients (531 alive and 187 dead) with somatic mutations were included in this study. We firstly investigated the genetic mutation landscape. The results of genetic mutation landscape in RCC patients with different vital states were shown in Figure 1A, 1C. In addition, we also investigated the mutation landscape of those 19 cuproptosis- related genes. ATP7B and NFE2L2 had 2% genetic mutation, ATP7A, GLS, MTF1, and DBT had 1% genetic mutation in alive RCC patients (Figure 1B). In dead RCC patients, the genetic mutation frequency of CDKN2A was 3%, NFE2L2 and NLRP3 were 2%, and ATP7A, ATP7B, GLS, DLAT, DLD, LIAS, PDHA1, and PDHB were 1% (Figure 1D). The TMB score in the RCC patients with alive was significantly higher than that in dead RCC patients (Figure 1E). However, there was no significant relationship between TMB and overall survival in patients with RCC (Figure 1F).

To obtain suitable cuproptosis-related biomarkers, we investigated the expression status of those 19 cuproptosis-related genes (CRGs) between alive and dead RCC patients. Only the expression level of the CDKN2A differed significantly between alive and dead RCC patients. CDKN2A was significantly increased in the dead RCC patients as measured by the following criteria basemean ≥ 50, Logfoldchange ≥ 0.5, and *p*adj < 0.05 (Figure 1G). The expression levels of the other 18 CRGsare displayed in Supplementary Figure 1. RCC patients with high expression of CDK2NA displayed worse OS (Figure 1H). Although CDKN2A was associated with OS in patients with RCC (Figure 1I), CDKN2A can’t be used as an independent OS-related biomarker for RCC as measured by the multivariate Cox regression analysis (Figure 1J).

### Independent prognostic biomarkers screening of RCC

**Figure 2:**
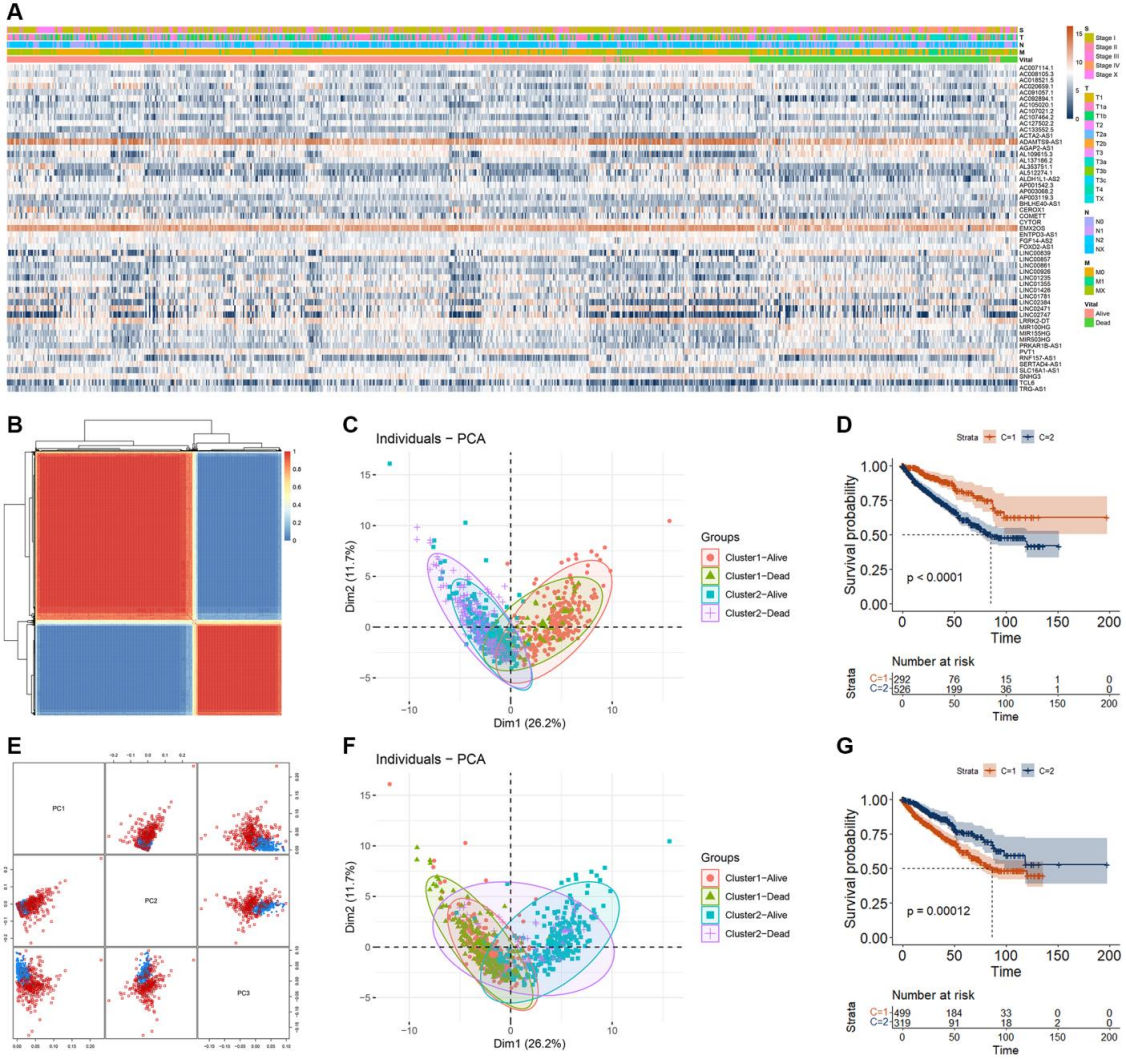
**RCC cluster analysis based on DECRLs.** (**A**) Heatmap of 53 DECRLs. (**B**) Cluster of RCC patients based on consensus analysis. (**C**) The distribution of RCC patients in different cluster (base on consensus analysis) and different survival status. (**D**) K-M curve of RCC patients with different cluster (base on consensus analysis). (**E**) Cluster of RCC patients based on MClust analysis. (**F**) The distribution of RCC patients in different cluster (base on MClust analysis) and different survival status. (**G**) K-M curve of RCC patients with different cluster (base on MClust analysis).

Cuproptosis was closely related to the cancer progression and may be a new therapeutic target for several cancers. To obtain suitable cuproptosis signatures as RCC biomarkers, we firstly performed the correlation analysis for those 19 CRGs and lncRNAs, and obtained 2872 cuproptosis-related lncRNAs (CRLs). Of those 2872 CRLs, a total of 53 CRLs were significantly different in expression between alive and dead RCC patients (28 CRLs were significantly increased and 25 CRLs were significantly decreased) (Figure 2A). RCC patients could be well divided into two clusters by using those 53 differentially expressed CRLs (DECRLs) as measured by consensusclust and MClust analysis (Figure 2B–2G). The survival curves of RCC patients in different cluster classified by these two methods were significantly different (Figure 2D, 2G).

**Figure 3:**
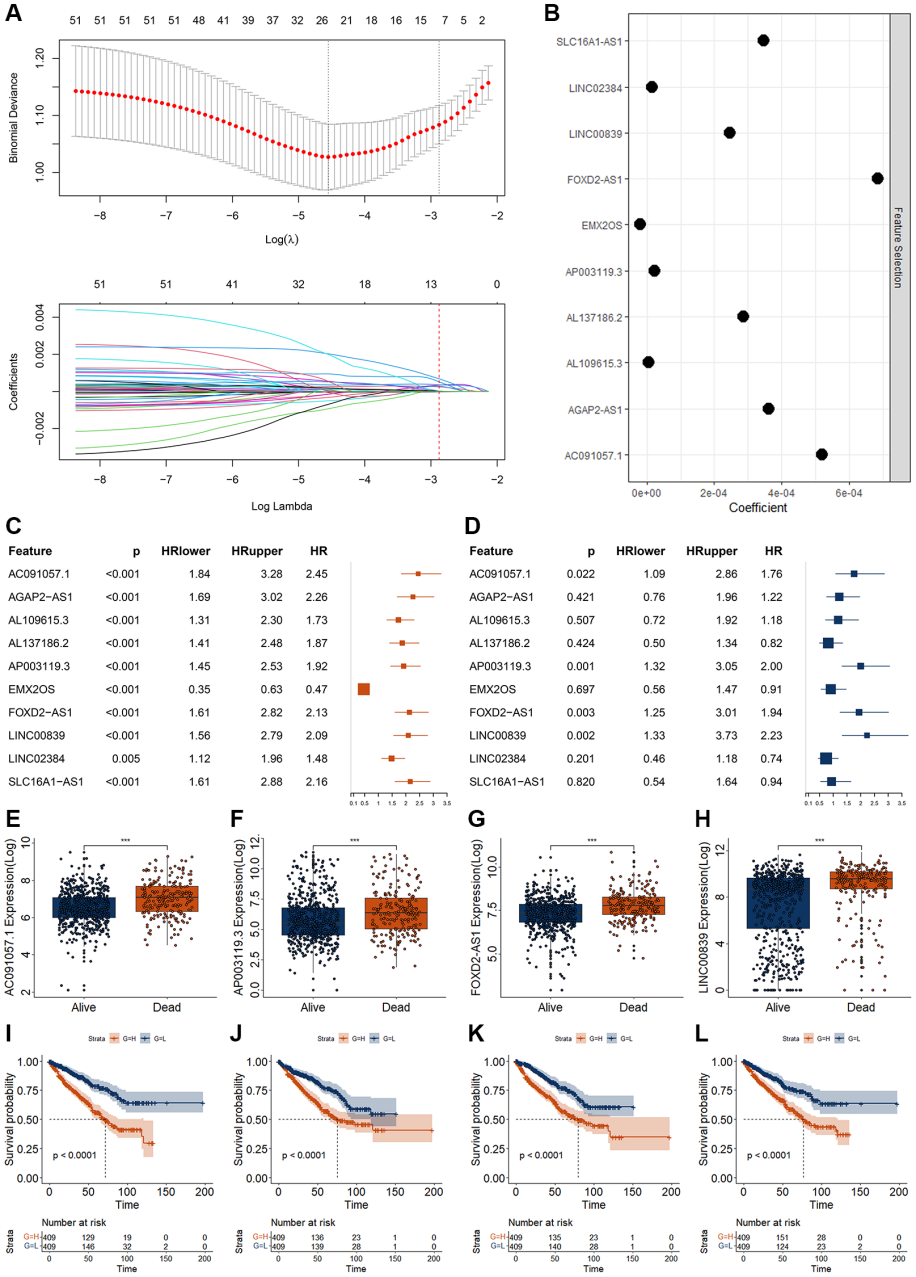
**Screening of RCC biomarkers based on DECRLs.** (**A**, **B**) Feature selection for 53 DECRLs using lasso algorithm. (**C**, **D**) Results of univariate (**C**) and multivariate (**D**) Cox regression for 10 DECRLs. (**E**–**H**) Expression of AC091057.1 (**E**), AP003119.3 (**F**), FOXD2-AS1 (**G**), LINC00839 (**H**) between alive and dead RCC patients. (**I**–**L**) K-M curve of AC091057.1 (**I**), AP003119.3 (**J**), FOXD2-AS1 (**K**), LINC00839 (**L**) in RCC patients. ^*^p < 0.05. ^**^p < 0.01. ^***^p < 0.001.

To screen out more suitable biomarkers from those 53 DECRLs, we first carried out feature selection analysis using lasso algorithm, and obtained 10 DECRLs, including AC091057.1, AGAP2-AS1, AL109615.3, AL137186.2, AP003119.3, EMX2OS, FOXD2-AS1, LINC00839, LINC02384, and SLC16A1-AS1 (Figure 3A, 3B). Then univariate and multivariate Cox regression were performed for those 10 DECRLs. All of those 10 DECRLs were significantly correlated with the OS of RCC patients (Figure 3C). To filter the OS independent correlated DECRLs, we divided RCC patients into training group and validation group randomly. Multivariate Cox regression analysis indicated that four DECRLs (AC091057.1, AP003119.3, FOXD2-AS1, and LINC00839) were independently correlated with the OS of RCC (Figure 3D). The expressions of those four DECRLs were significantly increased in RCC patient with dead status (Figure 3E–3H). Patients with high expression of those four DECRLs showed poor OS (Figure 3I–3L). RCC patients could also be well divided into two clusters by using those four DECRLs as measured by consensusclust and MClust analysis (Supplementary Figure 2).

### Construction and validation of the DECRLs-based prognostic model

**Figure 4:**
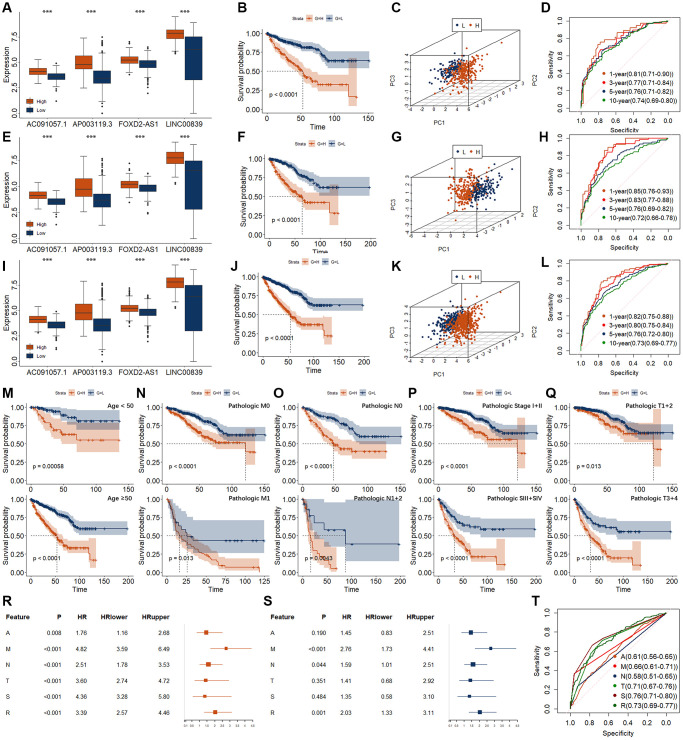
**Establishment and validation of risk models for RCC.** (**A**–**D**) Evaluation of risk model in training group, including expression level (**A**), K-M curve (**B**), PCA (**C**), and time-dependent ROC curve (**D**). (**E**–**H**) Evaluation of risk model in validation group, including expression level (**E**), K-M curve (**F**), PCA (**G**), and time-dependent ROC curve (**H**). (**I**–**L**) Evaluation of risk model in entire group, including expression level (**I**), K-M curve (**J**), PCA (**K**), and time-dependent ROC curve (**L**). (**M**–**Q**) K-M curves of risk models in entire group for different clinical phenotypes. (**M**) for age. Upper represents <50. Lower represents ≥50. (**N**) for pathologic M. Upper represents M0. Lower represents M1. (**O**) for pathologic n. Upper represents N0. Lower represents N1 + 2. (**P**) for pathologic T. Upper represents T1 + 2. Lower represents T3 + 4. (**Q**) for pathologic Stage. Upper represents SI + II. Lower represents SIII + IV. (**R**, **S**) Results of univariate (**R**) and multivariate (**S**) Cox regression for the risk model and different clinical feature. A, represents age. M, represents pathologic M. N, represents pathologic N. T, represents pathologic T. S, represents pathologic Stage. R, represents risk model. (**T**) ROC curve for the risk model and different clinical feature. ^*^p < 0.05. ^**^p < 0.01. ^***^p < 0.001.

According to previous studies, those four DECRLs (AC091057.1, AP003119.3, FOXD2-AS1, and LINC00839) were used to construct a prognostic model. Yonden index (Value = 20.565) from the training group was set as the optimal cutoff value (Supplementary Figure 3). All of those four DECRLs displayed increased level in RCC patients with high-risk value (Figure 4A). RCC patients with low-risk score exhibited better OS (Figure 4B). Principal component analysis (PCA) also showed RCC patients with low-risk score could be well distinguished from those patients with high-risk score (Figure 4C). All areas under curve (AUC) of time dependent receiver operating characteristic (ROC) curves were over 0.70 (Figure 4D). The one-year ROC curve value of the risk model even exceeds 0.80, reaching 0.81 (Figure 4D).

In the validation and entire group, we observed very similar results (Figure 4E–4L). In particular, we found that the AUC values of the 1-year and 3-year ROC curves in the validation group were as high as 0.85 and 0.83, respectively (Figure 4H). The AUC values of 1-year and 3-year ROC curves in the entire group were as high as 0.82 and 0.80, respectively (Figure 4L). Moreover, we found that the relationship between this risk model and survival was regardless of age, pathologic TNM, and Stage (Figure 4M–4Q).

High risk score was related with the metastasis of RCC (Table 2). Univariate and multivariate Cox regression analysis were performed for the risk model and different clinical features (Figure 4R–4T). Pathologic M and risk model could be the independent OS related signatures. However, the AUC value of risk (0.73) was higher than that of the pathologic M (0.66), little lower than that of the Stage (0.76).

**Table 2:** Correlation of risk model with pathologic N and M in RCC.

**Feature**		**Training**		**Validation**		**Entire**	
		**Low**	**High**	**Low**	**High**	**Low**	**High**
Pathologic N	No-	94	63	72	59	166	122
	Yes-	6	16	10	12	16	28
	χ^2^	8.317		0.685		6.975	
	*p*	0.004		0.408		0.008	
Pathologic M	No-	167	90	153	105	320	195
	Yes-	12	36	10	29	22	65
	χ^2^	26.665		15.503		41.187	
	*p*	<0.001		<0.001		<0.001	

In addition, we used machine learning to construct various types of prognostic prediction models using those four CRDELs (Supplementary Figure 4). We found that the risk model had the best AUC value.

### Assessment of immune landscape in RCC with different risk score

**Figure 5:**
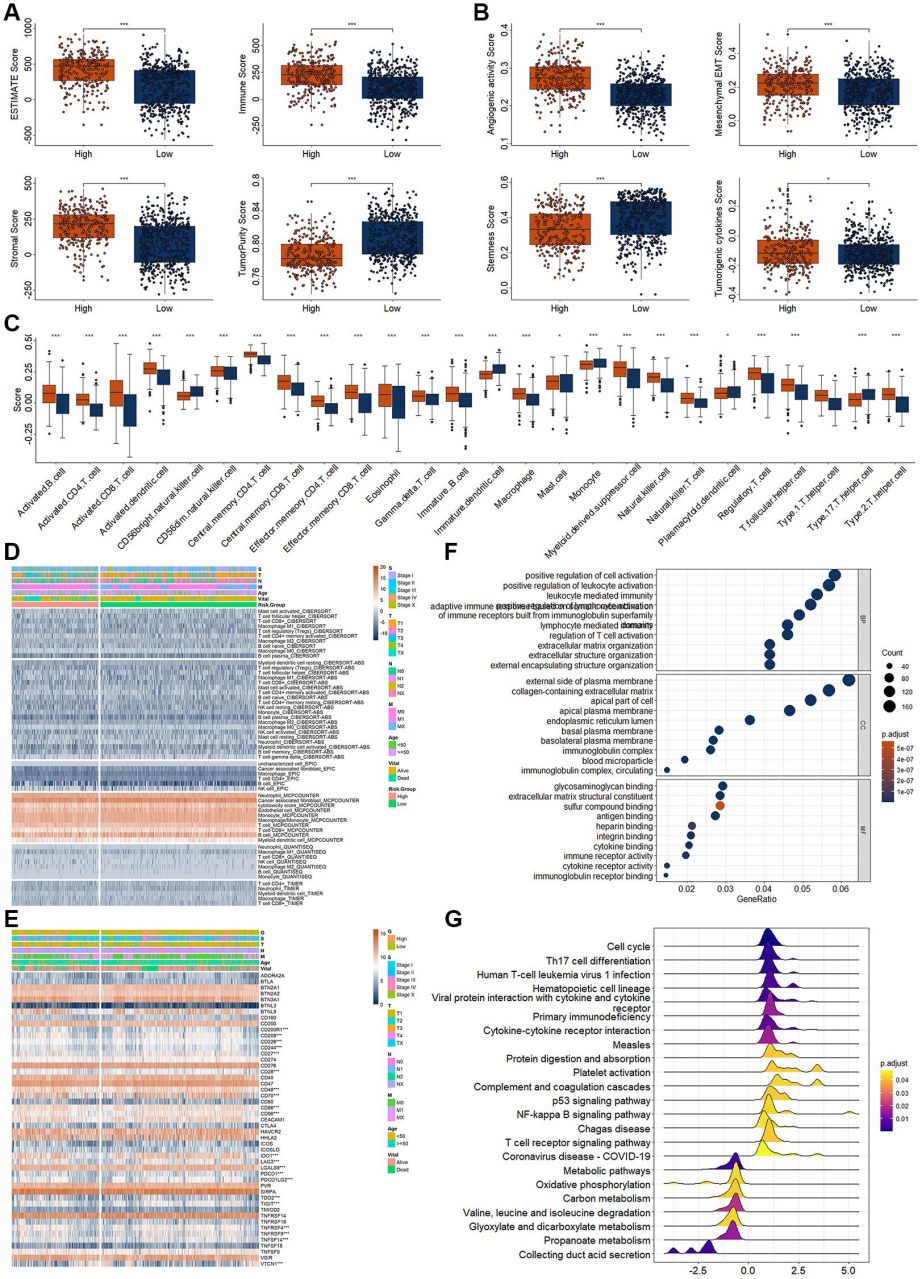
**Analysis of immunity in RCC patients based on risk model.** (**A**) Differential expression analysis of tumor microenvironment. (**B**) Differential expression analysis of tumor related score. (**C**) Differential expression analysis for the immune score of different immune cells and factors. (**D**) Differential expression analysis for the immune infiltration of different immune cells and factors. (**E**) Differential expression analysis for the immune checkpoint point genes between high- and low-risk score group. (**F**, **G**) Top 10 enriched GO (**F**) and KEGG terms (**G**). ^*^p < 0.05. ^**^p < 0.01. ^***^p < 0.001.

Previous study had demonstrated that cuproptosis could affect the immune response. Therefore, we analyzed the immune landscape between RCC patients with high and low-risk score. The ESTIMATE, immune, and stromal score were significantly increased, while the tumor purity score was significantly decreased in RCC patients with high-risk score (Figure 5A). The score of angiogenic activity, mesenchymal EMT, and tumorigenic cytokines were significantly higher in patients with RCC with high-risk score (Figure 5B). The score of stemness shows the opposite status (Figure 5B). We investigated the immune landscape of immune cells and factors, and found 26 of the 28 immune cells and factors differed significantly between the high- and low-risk RCC patients (Figure 5C). Of which, the immune scores increased significantly for 21 immune cells and factors and decreased significantly for five immune cells and factors in high-risk patients. To further clarify the relationship between immune landscape and risk model, we conducted correlation analysis for the immunity with those four DECRLs and risk model. The specific correlations were shown in Supplementary Figure 5.

Then, we also evaluated the infiltration landscape of different immune cells using different algorithm (Figure 5D). The infiltration values of many immune cells were significantly different, such as the cancer associated fibroblast, neutrophil, monocyte, macrophage/monocyte, and B cell by MCPCOUNTER algorithm (Figure 5D). These results indicated differences in immunity between high- and low-risk patients with RCC. Therefore, we proceeded to analyze differences in immune-related genes expressions between high- and low-risk patients. The results as shown in Figure 5E, the expression of several immune-related genes differed significantly between high and low risk groups (Figure 5E).

Additionally, enrichment of GO and KEGG analysis also indicated that several immune related terms had been enriched, such as positive regulation of cell activation, regulation of T cell organization, extracellular matrix organization in GOBP, Th cell differentiation, cytokine-cytokine receptor integration, T cell receptor signaling pathway in KEGG (Figure 5F, 5G).

### Evaluation of immune response and chemotherapy response base on risk model

**Figure 6:**
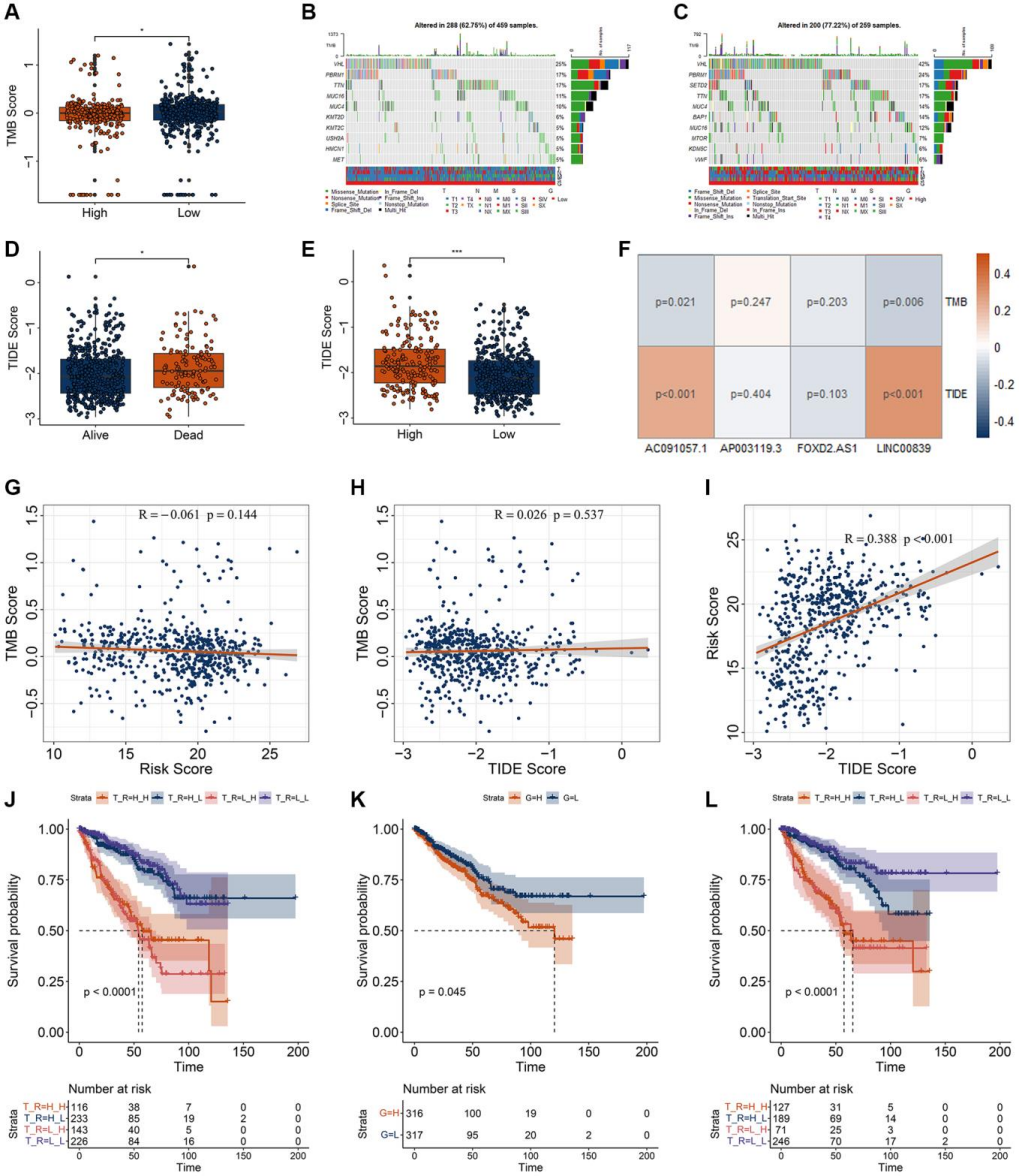
**Correlation analysis for the TMB and TIDE with risk model.** (**A**) Differential analysis of TMB for RCC patients with different risk score. (**B**, **C**) genetic mutation of RCC patients with low- (**B**) and high-risk (**C**) score. (**D**, **E**) Differential analysis of TIDE for RCC patients with different vital status (**D**) and risk score (**E**). (**F**) Correlation of four DECRLs with the TMB and TIDE score. (**G**–**I**) Correlation analysis of TMB, TIDE, and risk score. (**G**) Correlation of risk score with TMB score. (**H**) Correlation of TIDE score with TMB score. (**I**) Correlation of risk score with TIDE score. (**J**) K-M curve for RCC patients with different TMB and risk score. H_H, represents high TMB + high risk. H_L, represents high TMB + low risk. L_H, represents low TMB + high risk. L_L, represents low TMB + low risk. (**K**) K-M curve for RCC patients with different TIDE. H, represents high TIDE. L, represents low TIDE. (**L**) K-M curve for RCC patients with different TIDE and risk score. H_H, represents high TIDE + high risk. H_L, represents high TIDE + low risk. L_H, represents low TIDE + high risk. L_L, represents low TIDE + low risk. ^*^p < 0.05. ^**^p < 0.01. ^***^p < 0.001.

Previous studies indicated the genetic mutations were correlated with the immune response. Immunotherapy response was measured using TMB and TIDE score. Therefore, we re-investigated the genetic mutations in RCC patients with high- and low-risk score. RCC patients with low-risk score had higher TMB score (Figure 6A). In the low-risk group, the top 10 mutations were VHL (25%), PBRM1 (17%), TTN (17%), MUC16 (11%), MUC4 (10%), KMT2D (6%), KMT2C (5%), USH2A (5%), HMCN1 (5%), and MET (5%) (Figure 6B). In the high-risk group, the top 10 mutations were VHL (42%), PBRM1 (24%), SETD2 (17%), TTN (17%), MUC4 (14%), BAP1 (14%), MUC16 (12%), MTOR (7%), KDM5C (6%), and VWF (6%) (Figure 6C). K-M curve showed survival in high - and low-risk patients was not affected by TMB. Compared to the alive RCC patients, the TIDE scores were dramatically higher in the dead RCC patients (Figure 6D). Compared to the low-risk group, the TIDE scores were dramatically higher in the high-risk group (Figure 6E). The correlation of those four DECRLs and risk score with the TMB and TIDE score were displayed in Figure 6F–6I. The risk score was significantly correlated with the TIDE score (Figure 6I). Low-TMB RCC patients and high-TMB RCC patients showed comparable survival rates (Figure 2F). When combined risk values were analyzed, high-risk and low-risk patients showed significantly different survival rates (Figure 6J). Moreover, we also found that RCC patients with high TIDE score exhibited worse OS (Figure 6K). When TIDE was combined with risk, we found that patients with low TIDE and low risk score showed the best prognosis (Figure 6L).

**Figure 7:**
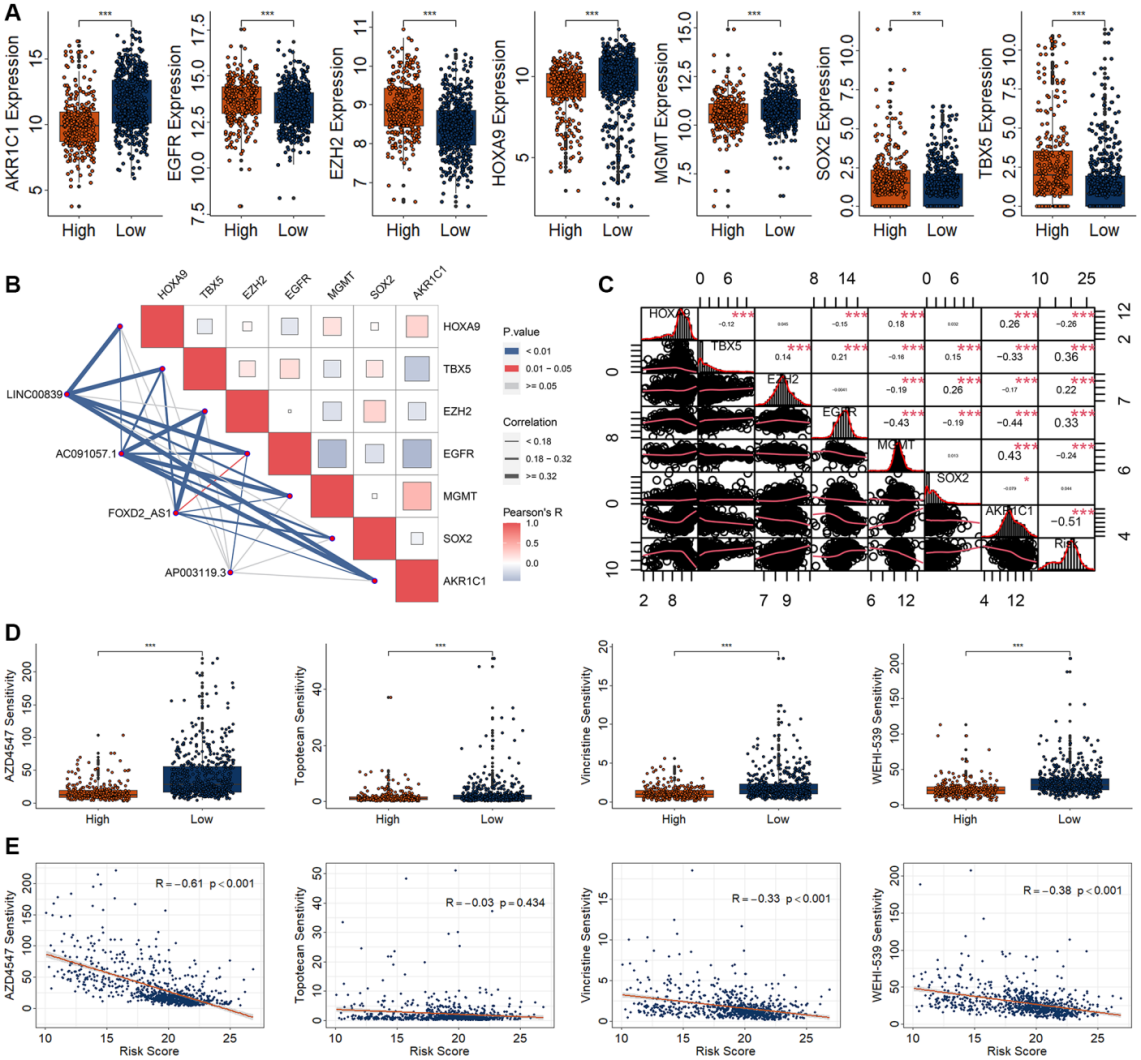
**Drug sensitivity analysis in patients with RCC based on a risk model.** (**A**) Differential expression analysis of seven CRSGs between high- and low-risk group. (**B**) Correlation analysis for the four DECRLs with seven CRSGs. (**C**) Correlation analysis for the risk model with seven CRSGs. (**D**) Differential analysis of four kinds of drug sensitivity. (**E**) Correlation analysis for those four drugs with the risk score. ^*^p < 0.05. ^**^p < 0.01. ^***^p < 0.001.

To determine chemotherapy drug sensitivity among high- and low-risk group, we firstly observed the expression of several chemoradiotherapy sensitivity–related genes (CRSGs), including AKR1C1, EGFR, EZH2, HOXA9, MGMT, SOX2, and TBX5 [24]. The expression of AKR1C1, HOXA9, and MGMT were significantly increased in low-risk group, while the expression of EGFR, EZH2, SOX2, and TBX5 were significantly decreased in high-risk group (Figure 7A). These CRSGs were correlated with candidate biomarkers and risk models (Figure 7B, 7C).

Oncoppredict algorithm is used to evaluate the sensitivity of different chemotherapy agents by screening the differentially expressed genes obtained in high-risk and low-risk RCC groups. In order to screen and obtain more sensitive drugs, we set the IC50 of the drug to be less than 50, LogFC>|0.5|, and *p* < 0.05. Out of 177 candidates, we obtained 19 candidates’ drugs, including AZD4547, AZD3759, AZD5153, AZD7762, Dasatinib, Erlotinib, Gefitinib, IGF1R_3801, IWP-2, MK-8776, OTX015, Palbociclib, Sapitinib, Sepantronium bromide, Topotecan, VE-822, Vincristine, Wee1 Inhibitor, and WEHI-539 (Supplementary Figure 6). Among them, the more sensitive drugs for the RCC patients with high-risk score were AZD4547, Topotecan, Vincristine, and WEHI-539 (Figure 7D). Moreover, AZD4547, Vincristine, and WEHI-539 were negatively correlated with the risk score (Figure 7E).

## DISCUSSION

Kidney cancer is the second most common cancer of the urinary system. Distant metastases occur in about 40% of patients with advanced kidney cancer, with a poor 5-year prognosis of about 10% [18]. It is of great significance to construct a suitable risk model for accurate prognosis and clinical medication guidance. Cuprorptosis is a novel pathway of cell death, which is distinguishable from apoptosis, necroptosis, pyroptosis, and ferroptosis [25]. Cuproptosis provides a new sight in disease treatment, including cancers [26, 27]. In the present study, we carried out compressive analysis and obtained four DECRLs could be the prognostic biomarkers for RCC, including AC091057.1, AP003119.3, FOXD2-AS1, and LINC00839. The constructed risk model using those four CRDELs could be an independent overall survival related signature, and well predict the outcome of RCC patients as measured by AUC value. Results from Jiang and Jin et al. demonstrated that high expression of AC091057.1 was positive correlated worse OS in pancreatic cancer and lung cancer respectively [28, 29]. In our present study, we also found that RCC patients with high expression of AC091057.1 exhibited worse OS. Li et al. found that AP003119.3 expression was positive correlated with the OS, and could be a prognostic biomarker for breast cancer [30]. Several studies showed FOXD2-AS1 was associated with many cancers, such as breast cancer, ovarian cancer, retinoblastoma, and cervical cancer. High expression of FOXD2-AS1 could promote the progression of cancer cell, including proliferation, migration, and invasion [31–35], and could be prognostic biomarker for many cancers, such as oral squamous cell carcinoma, head and neck squamous cell carcinoma, renal cancer [36–38]. LINC00839 could promote the progression of several cancers, including gastric cancer, neuroblastoma, colorectal cancer, and liver cancer [39–42]. Consistence with previous studies, we found the expressions of those four DECRLs were significantly increased in RCC patients with dead status. Moreover, their high expression was strongly associated with poor survival. All of those results reinforced the relationship of AC091057.1, AP003119.3, FOXD2-AS1, and LINC00839 with cancers.

In the past, many researchers carried out studies on the prognostic model construction of RCC. In our previous study, we found Klotho and Sortilin 1 were significantly correlated with the OS of RCC. The 1-year AUC value of the risk model using those two biomarkers reached 0.7 [43]. Zhao et al. found six-snoRNA signatures (SNORA70B, SNORD12B, SNORD93, SNORA59B, SNORD116-2, and SNORA2) were superior indicators to routine clinical factors (AUC = 0.732) [44]. Li et al. found that patients with low PIK3CA expression had poorer overall survival, and the AUC of their ROC curve was 0.775 [45]. Li et al. found that the area under the curve (AUC) values of 1-year, 3-year and 5-year survival rates of the model constructed with seven cuproptosis-related genes were 0.814, 0.762 and 0.825, respectively [46]. Li et al. constructed six-gene biomarkers (ARPC3, PHF19, FKBP11, MS4A14, KDELR3 and CD1C) to predict the 1-year, 3-year and 5-year efficacy of 0.911, 0.845 and 0.867 (AUC), respectively. Comparatively, the AUC values of 1-year, 3-year, 5-year, and 10-year ROC curves of the risk model constructed with four cuproptosis-related markers were 0.82, 0.80, 0.76, and 0.73 in this study respectively. This model can achieve higher AUC value with fewer biomarkers.

Understanding the immune status and tumor microenvironment of tumor patients is of great significance for the treatment of tumor patients. In this study, we found that stomal score, immune score, and ESTIMATE score were significantly higher in high-risk RCC patients, and tumor purity were significantly lower in high-risk RCC patients. Tumor purity refers to the proportion of tumor cells in the tumor tissue. Tumor tissue includes not only tumor cells, but also many other cells associated with the tumor microenvironment, such as immune cells. ESTIMATE is a tool that uses genes expression data to predict tumor purity and the presence of stromal and immune status in tumor tissue [47, 48]. Tumor samples with lower tumor purity have more immune cells, and the mutation load is often higher, because the inflammatory response caused by immune cells can increase the mutation rate of tumor cells, and the immunotherapy effect may be better [48]. In the evaluation studies of tumor microenvironment, we found that the scores of angiogenic activity, mesenchymal EMT, and tumorigenic cytokines were significantly increased in high-risk RCC patients, and stemness score was significantly decreased in high-risk patients. Tumor stem cells are cancer cells that have characteristics associated with normal stem cells and can produce all cell types in a specific cancer sample [49]. Such cells are generally thought to have the potential to form tumors and develop into cancer, especially as the cancer metastasizes, giving rise to new types of cancer [49]. In this study, we found that increased dry index scores in low-risk RCC patients suggest that low-risk RCC patients are at risk for further deterioration.

In relevant studies on immunotherapy, our model suggests that this risk value is significantly correlated with the response to immunotherapy, which further confirms the relationship between cuproptosis and immunity, and also provides a new insight for the involvement of cuproptosis in the treatment of kidney cancer [50–52]. In the relevant studies on the sensitivity of chemotherapy drugs, we found that three drugs (AZD4547, Vincristine, and WEHI-539) were highly sensitive to high-risk RCC patients and may be used in the clinical treatment of high-risk RCC patients. AZD4547 can inhibit the cell growth of several cancer cells, such as head and neck cancer, breast cancer, and colorectal cancer [53–55]. Vincristine has been utilized in several polytherapy regimens for acute lymphoblastic leukemia, neuroblastoma and rhabdomyosarcoma [56, 57]. Choiou et al. found WEHI-539 and ABT-199 coordinately promote degradation of MCL1 in human leukemia cells [58]. Abed et al. found that WEHI-539 and ABT-737 had a synergistic effect with carboplatin to enhance cell death in cell growth tests [59]. In our study, we found that AZD4547, Vincristine, and WEHI-539 may be used in the treatment of high-risk RCC patients, and these studies further expand their use in cancer.

## CONCLUSION

In this study, we identified four candidate biomarkers through a combined analysis. Four DECRLs (AC091057.1, AP003119.3, FOXD2-AS1, and LINC00839) were able to better predict the prognosis of patients with RCC. Furthermore, we found that this model was significantly associated with immune response and multi-drug sensitivity, suggesting that this model can be used to guide clinical applications of RCC, including immunotherapy and chemotherapy.

## Supplementary Materials

Supplementary Figures
