# Supplementary material for: Characterization of tumor microenvironment and sensitive chemotherapy drugs based on cuproptosis-related signatures in renal cell carcinoma
Source: Aging (Albany NY). 2023 Sep 19;15(18):9695–717. doi: 10.18632/aging.205043 (PMC10564438; doi:10.18632/aging.205043)
Supplement: Supplementary Figures [file aging-15-205043-s001.pdf]

SUPPLEMENTARY FIGURES

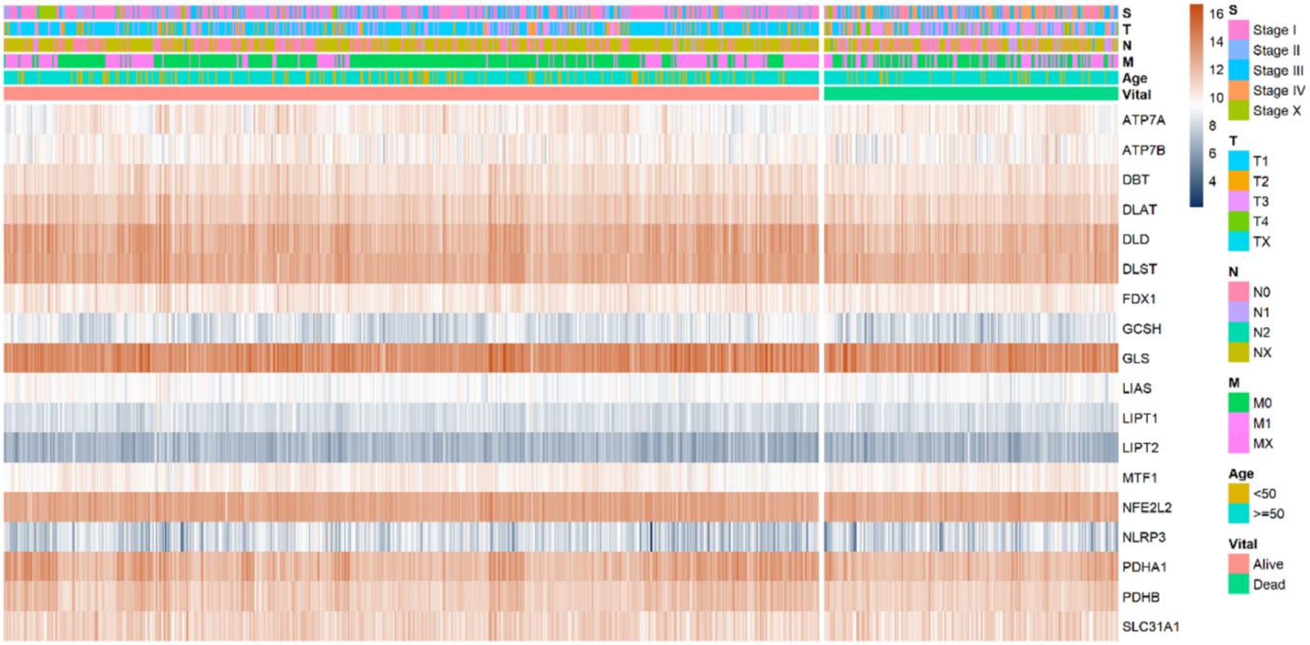

Supplementary Figure 1. Expression heat map of 18 genes related to cuproptosis.

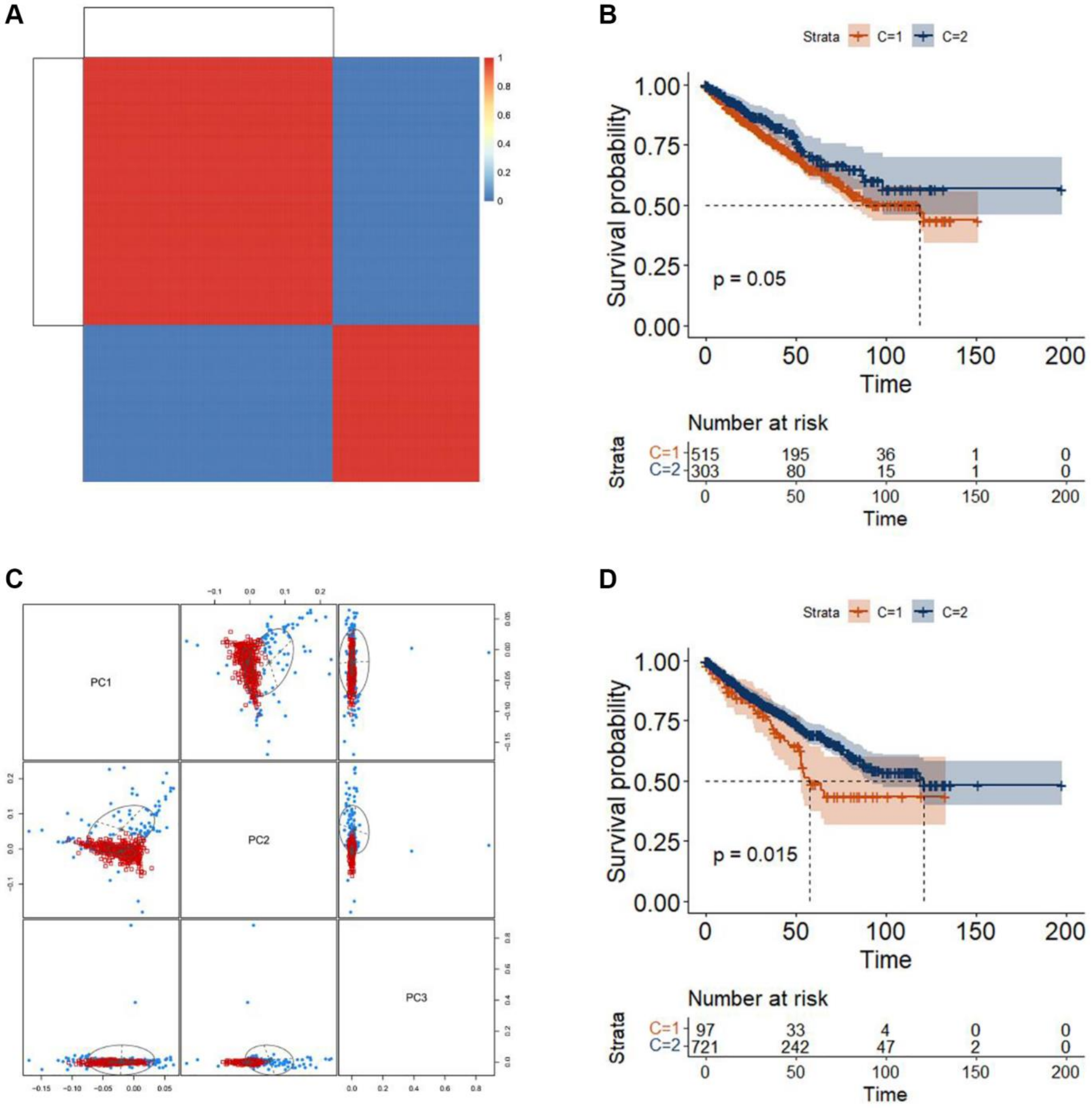

Supplementary Figure 2. Consensus and MClust analysis based on four DECRLs. (**A**) Cluster of RCC patients based on consensus analysis. (**B**) K-M curve of RCC patients with different cluster (base on consensus analysis). (**C**) Cluster of RCC patients based on Mclust analysis. (**D**) K-M curve of RCC patients with different cluster (base on MClust analysis).

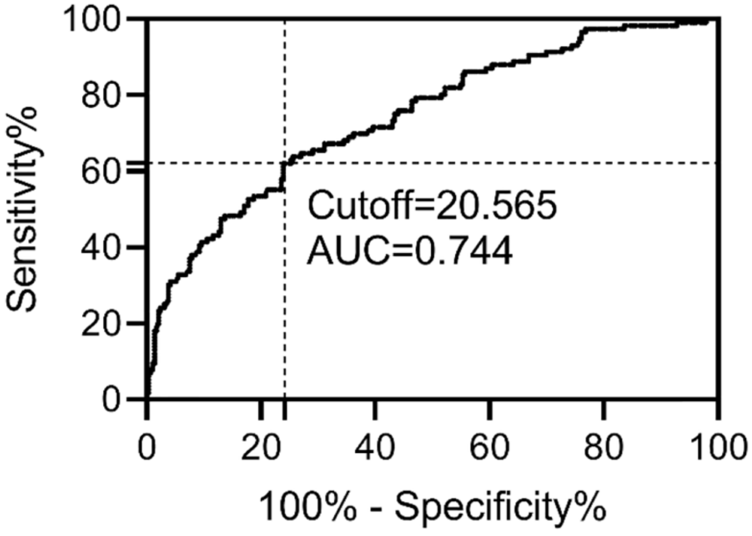

Supplementary Figure 3. Cutoff value from the risk model in the training group.

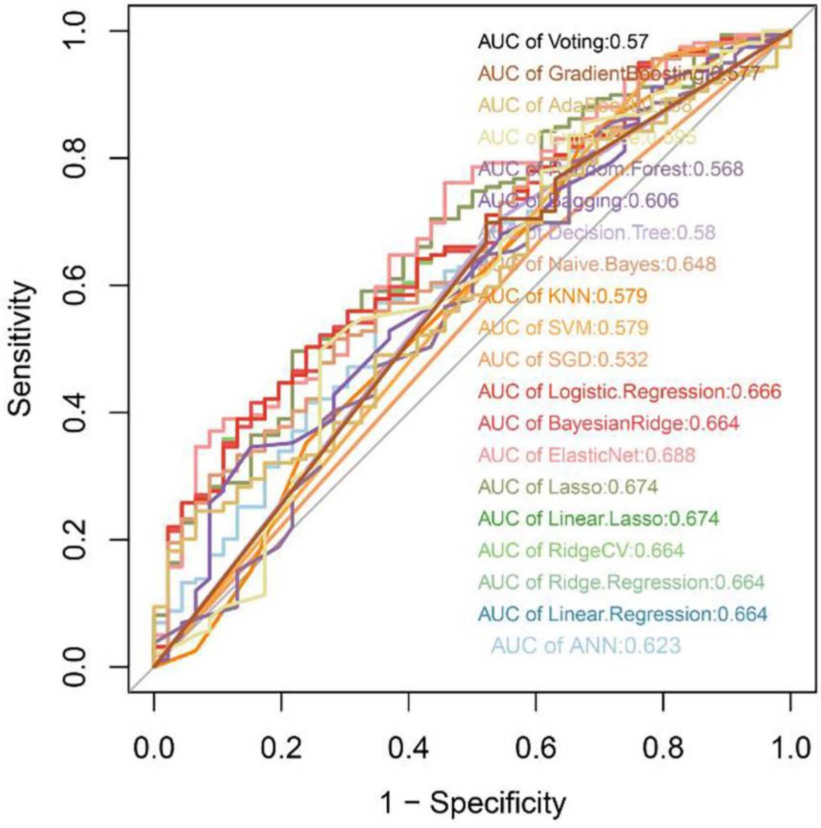

Supplementary Figure 4. Various types of prognostic prediction models using those four CRDELs based on machine learning analysis.

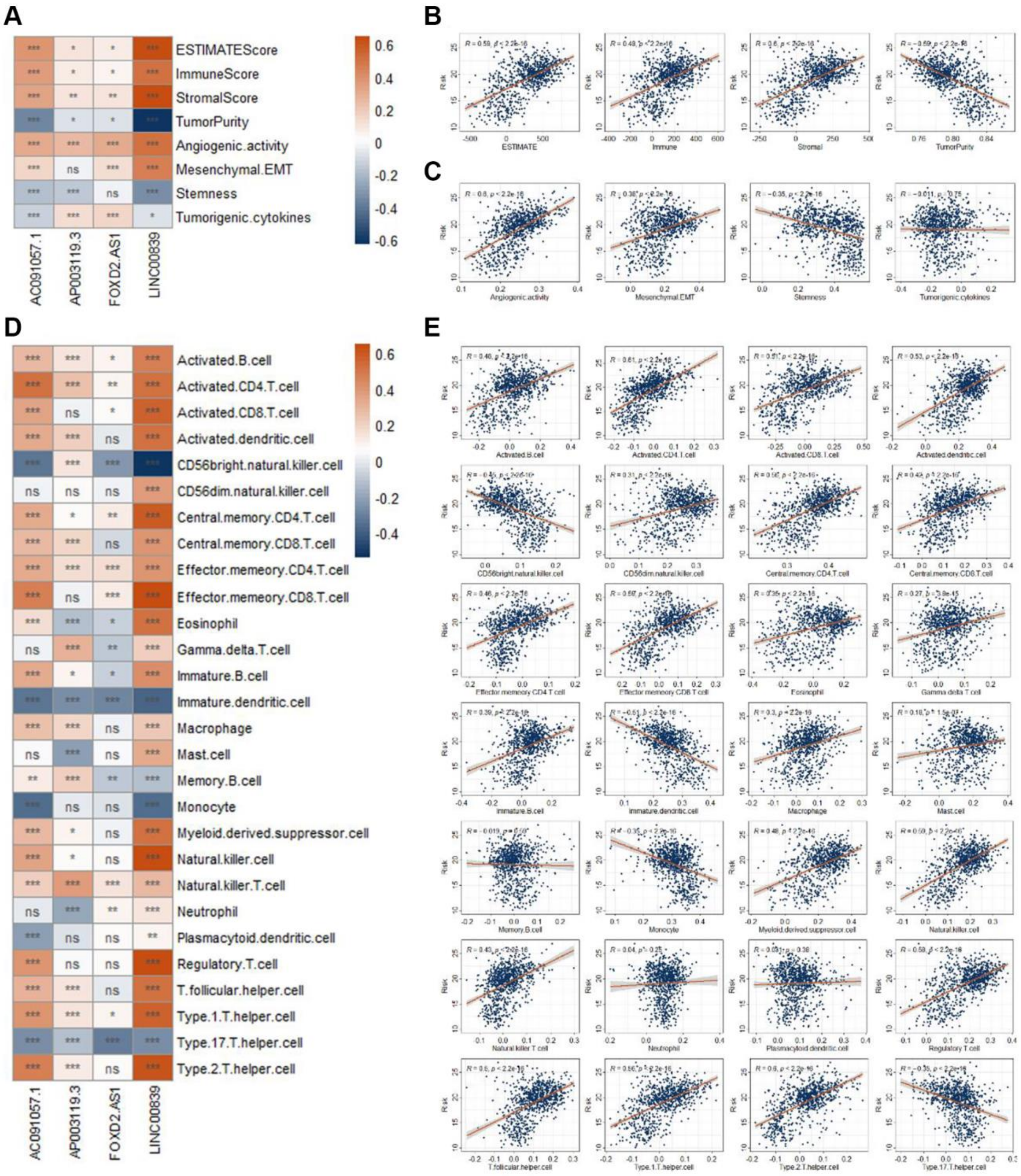

Supplementary Figure 5. Correlation analysis for the immunity with four DECRLs and risk model. (**A**) Correlation analysis of tumor microenvironment and tumor related score with four DECRLs. (**B**, **C**) Correlation analysis of tumor microenvironment (**B**) and tumor related score (**C**) with risk model. (**D**) Correlation analysis of immune score of different immune cells and factors with four DECRLs. (**E**) Correlation analysis of immune score of different immune cells and factors with risk model.

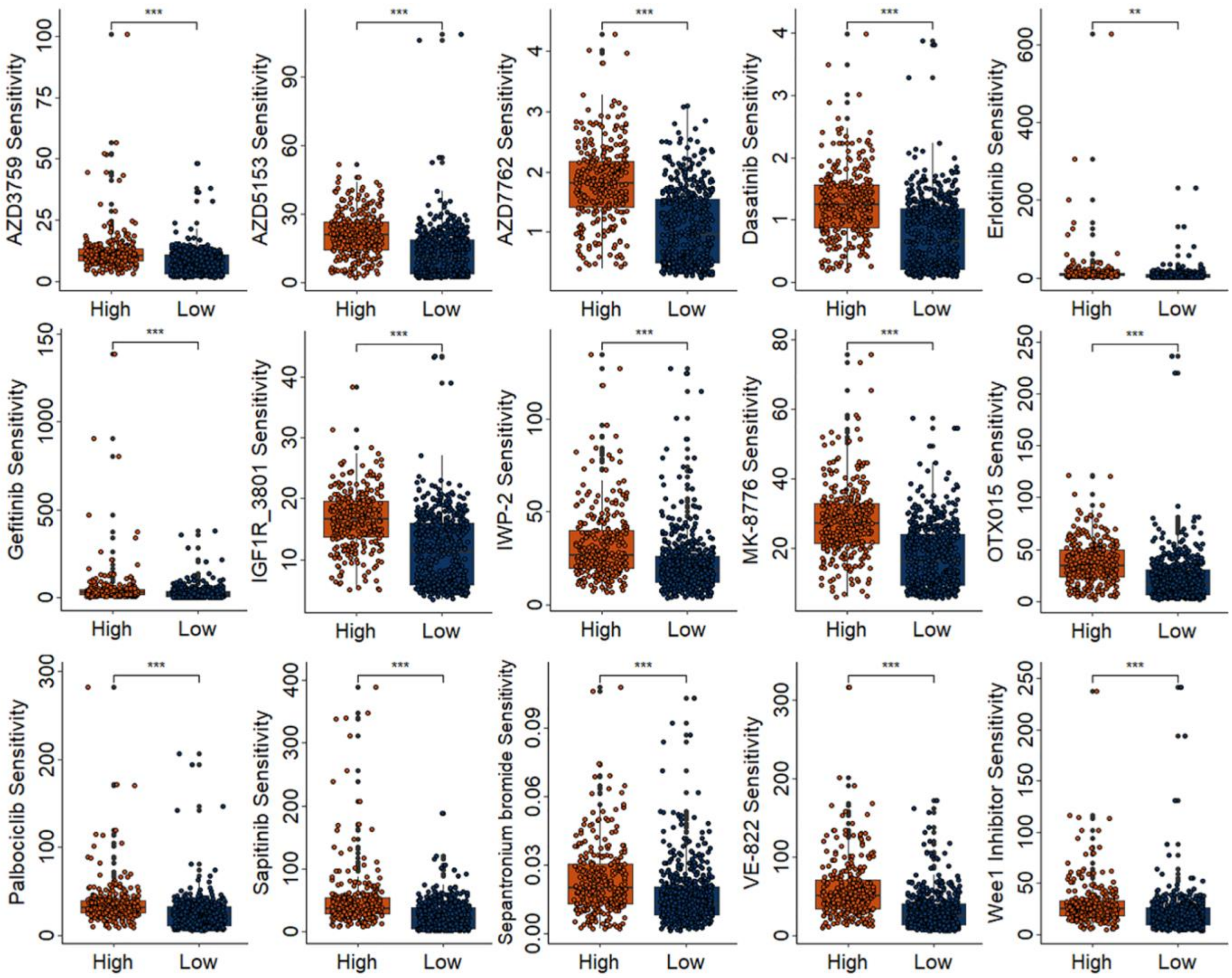

Supplementary Figure 6. Differential analysis of drug sensitivity for the 15 drugs.
